# A Lumpy Bumpy Liver

**DOI:** 10.14740/gr676w

**Published:** 2015-10-21

**Authors:** Sunil V. Pawar, Vinay G. Zanwar, Samit S. Jain, Pravin M. Rathi

**Affiliations:** a Department of Gastroenterology, 7th Floor OPD Building, Topiwala National Medical College and Bai Yamunabai Laxman Nair Hospital, Mumbai Central, Mumbai, Maharashtra, India

## To the Editor

**Figure 1:**
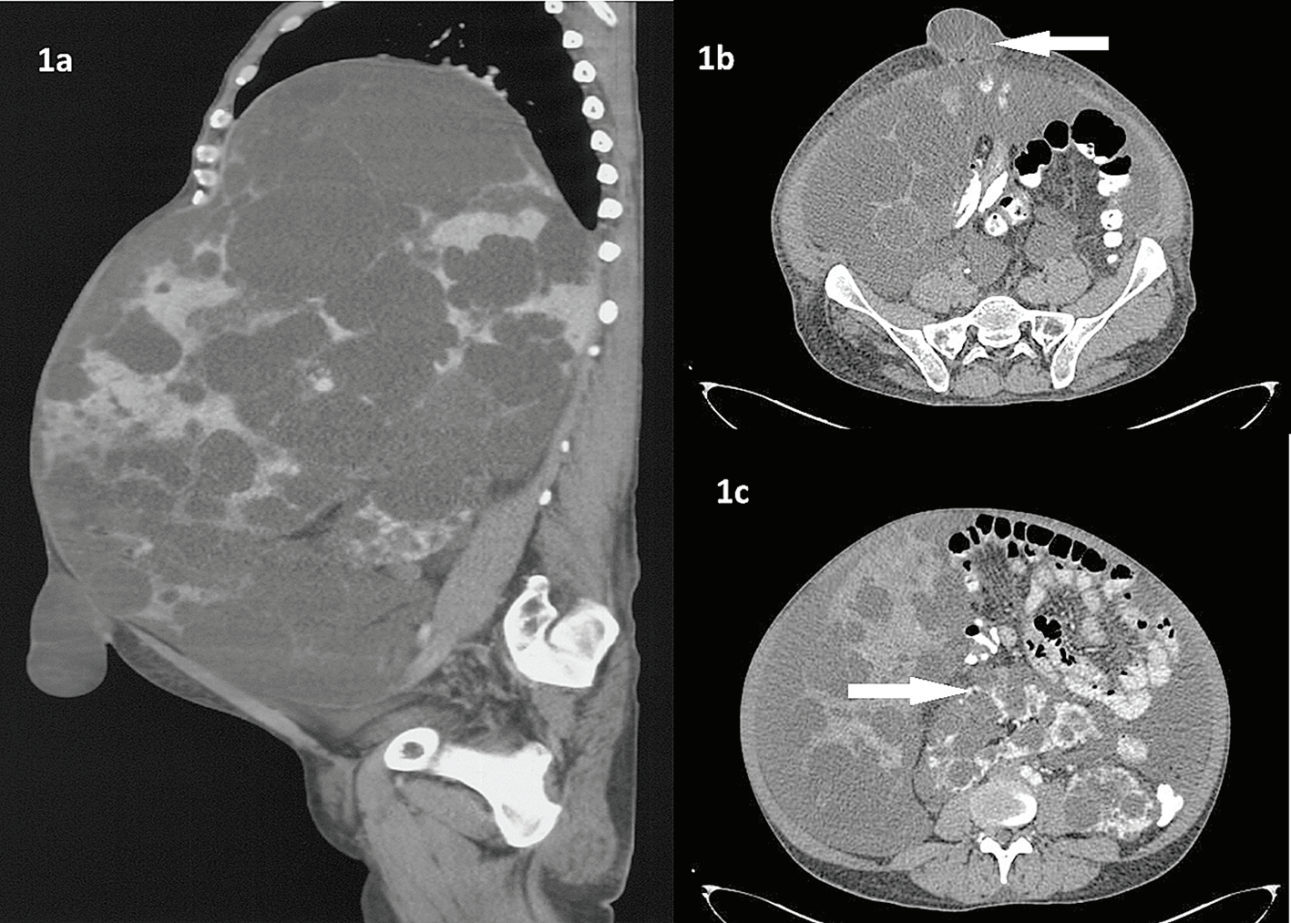
(a) Massive hepatomegaly with cystic lesions with umbilical hernia. (b) Umbilical hernia with hepatic cyst as its content. (c) Bilateral kidneys showing irregular lobulated contour with cystic lesions. Also note malrotation of right kidney.

A 40-year-old male patient presented with distension of abdomen since last 4 years. The distension was progressive and more in upper abdomen. He also developed umbilical hernia since 1 year. There was no jaundice or bleeding. On examination, massive hepatomegaly till right iliac fossa with multiple nodular swellings was felt. The computed tomography of abdomen showed hepatomegaly of 36 cm in size reaching up to pelvis (Fig. 1a). There were multiple variable sized non-enhancing hypodense cystic lesions seen in liver. A 3.4 × 3.5 cm defect was seen in anterior abdominal wall in umbilical region with liver cysts as its content (Fig. 1b). Right kidney was seen in midline in the pelvis facing posteriorly. Both kidneys were enlarged with irregular lobulated contour with cystic lesions (Fig. 1c). The free fluid is seen in abdomen and pelvis. This is a case of polycystic liver and kidney disease with portal hypertension with ascites. Ultrasonography of the family members were normal. The patient was started on salt restricted diet and diuretics. The umbilical hernia was reduced and abdominal binder was applied.

The polycystic liver disease occurs as an extra-renal organ involvement in polycystic kidney disease. Rarely its presentation can be isolated. The cystic transformation of liver is secondary to ductal plate malformation and abnormal fluid secretion by cholangiocytes [1]. This can be managed with medications and genetic counseling. The surgical options are fenestration but recurrence is high, segmental hepatic resection if the cysts are localized and liver transplantation with or without kidney transplantation [2].
